# Relationship between Salivary Alpha-Amylase Enzyme Activity, Anthropometric Indices, Dietary Habits, and Early Childhood Dental Caries

**DOI:** 10.1155/2022/2617197

**Published:** 2022-03-26

**Authors:** Zahra Parsaie, Peyman Rezaie, Niloofar Azimi, Najmeh Mohammadi

**Affiliations:** ^1^Department of Pediatric Dentistry, Zahedan Dental School, Zahedan University of Medical Sciences, Zahedan, Iran; ^2^Department of Pediatric Dentistry, Shiraz Dental School, Shiraz University of Medical Sciences, Shiraz, Iran; ^3^Adelaide Medical School and Centre of Research Excellence in Translating Nutritional Science to Good Health, Faculty of Health and Medical Sciences, University of Adelaide, Adelaide 5005, Australia; ^4^Oral and Dental Disease Research Center, Shiraz Dental School, Shiraz University of Medical Sciences, Shiraz, Iran

## Abstract

**Objectives:**

Although early childhood dental caries (ECC) have the same general etiology as other types of caries, predisposing factors are not well elucidated. This study aimed to investigate the effect of salivary alpha-amylase (sAA) activity, body mass index (BMI), dietary habits, and oral hygiene on ECC.

**Methods:**

This cross-sectional study was performed on 38 ECC-affected and 41 caries-free children, aged 36 to 72 months. Upon the parents' consent, 3 mL of non-stimulated saliva was collected from the participants to measure the level of sAA activity through spectrophotometry. Additionally, parents/caretakers completed a structured questionnaire about demographic factors, oral hygiene, and consumption of sugar-containing foods. BMI, BMI z-scores, and percentile data were calculated by using an online calculator. The independent variables were dichotomized and tested through chi-square test, followed by a stepwise logistic regression, by using SPSS software (*α* = 0.05).

**Results:**

The sAA activity was significantly higher in caries-free children (*P* ≤ 0.001). However, the mean BMI was not significantly different between the two groups (*P*=0.49). Brushing and other measured dietary habits were significantly associated with the development of ECC (*P* ≤ 0.001). According to the results of the logistic regression, sAA activity was shown to be a predictor for ECC development (*Odds ratio (95% confidence interval)*: 0.9 (0.95–0.98)).

**Conclusion:**

Children with inherently lower levels of sAA activity were more susceptible to dental caries. Improper nutritional habits and poor oral health care could exacerbate the risk of ECC.

## 1. Introduction

Predicting caries incidence in young children is becoming increasingly important due to the health care costs and resources constraints [1]. According to the American Academy of Pediatric Dentistry, early childhood caries (ECC) is an important chronic disease, which is progressively caused by the imbalance of various risk and protective factors [2]. This oral health problem is more prevalent among socially deprived communities [3]. It includes the presence of one or more decayed (non-cavitated or cavitated lesions), missing (due to caries), or filled tooth surfaces of any primary tooth in children ≤71 months [2]. The general etiology of ECC is the same as general dental caries (microbiological, dietetic, and environmental risk factors); however, the predisposing factors are poorly identified [4].

Severe ECC is said to be the result of a combination of parents' socioeconomic, psychological, and behavioral features [5]. Its high prevalence is chiefly due to incorrect feeding practices, socioeconomic status of the family, parental illiteracy or inadequate education, and inaccessibility of dental care [3]. However, it can be prevented through establishing healthy eating habits, efficient brushing and flossing, receiving regular preventive dental services, and community-based educational programs [6].

Recently, attentions have been attracted to the possible association between the body composition and dental caries in children [7–12]. Evidence shows that obesity and dental caries hold the two risk factors of dietary features and socioeconomic status in common [13]. However, contrasting reports exist regarding the association between dental caries and body mass index (BMI) as the measurement of body fatness, particularly in younger children [9, 13–16].

On the other hand, the physical features and chemical structure of saliva are reported to have imperative protective mechanisms against dental caries [17], so that some salivary proteins can act as key biomarkers for dental caries [18]. The most abundant enzyme in human saliva is the salivary alpha-amylase (sAA) [19], which hydrolyzes the starch to glucose and maltose and helps food digestion [20]. This salivary enzyme of the acquired enamel pellicle regulates bacterial colonization and adds glucose for biofilm formation. On the other hand, this protein binds to the membrane of cariogenic bacteria and facilitates their elimination from the oral cavity through the salivary clearance, and consequently reduces the risk of dental caries [21].

Considering the fact that the amylase gene (*AMY1*) shows extensive copy number variations which are directly proportional to the salivary *α*-amylase content and activity in saliva [22], evaluation of this enzyme could be a helpful method for determining individuals with high risk of dental caries [23]. Confirming this issue, an association has been reported between some amylase gene (*AMY1*) copy number variations and high occurrence of smooth-surface caries [24].

Although, high sAA concentration in saliva was found to be positively related with more dental caries incidence in some studies [20, 25–28], controversies exist regarding the relationship between the concentration, function, or activity of this protein in saliva in this regard [21, 29].

Given the inconsistency and restriction of data about the predictors of ECC, this study aimed to investigate the effect of salivary alpha-amylase (sAA) activity, body mass index (BMI), dietary habits, and oral hygiene on the ECC development.

## 2. Materials and Methods

### 2.1. Study Population

This study was ethically approved by the Ethics Committee of Shiraz School of Dentistry (IR.SUMS.DENTAL.REC.1399.037). All methods were performed in accordance with the relevant guidelines and recognized ethical standards of Shiraz University of Medical Sciences (Ethics Committee) in order to protect the rights, safety, and dignity of all participants. Written informed consent was obtained from the participants' parents/guardians after explaining the aims and methods of the study. A total number of 200 children (100 girls and 100 boys, 36 to 72 months old) referring to the Department of Pediatric Dentistry of Shiraz School of Dentistry, from September to December of 2020 were screened. The children who were older than 72 months, uncooperative in collecting saliva, unable to expectorate, and those with no teeth, congenital or systemic disease, and a history of extensive dental interventions were excluded. Finally, 38 caries-active (ECC) and 41 caries-free (control) children, matched with respect to the mentioned criteria, selected by simple random sampling, remained in the study.

### 2.2. Experimental Design

Dental caries was diagnosed and confirmed through visual inspection according to the World Health Organization criteria. A single calibrated examiner dried the teeth by using a sterile gauze and explored caries with a dental mouth mirror under artificial light. Periodontal probe and dental floss were used to remove dental plaque present on the surfaces. The examiner recorded whether the surfaces were sound, decayed, filled, or indicated for extraction [30]. The intraexaminer agreement was calculated (Kappa = 0.8).

Saliva samples were collected on a different day from dental examination. We recommended to the parents to perform the regular oral hygiene procedure for children, after breakfast. Children were prevented from eating or drinking until sample collection.

To collect 3 mL of non-stimulated saliva through spiting, the child was asked to sit on a chair and lean forward while dropping down the head. Since the level of salivary secretion might be different during the day, all samples were collected between 9 and 11 a.m. within a maximum procedure of 30 minutes. The saliva samples were stored at 4 C and then transferred to the laboratory.

### 2.3. Alpha-Amylase Activity Assay

The saliva samples were centrifuged and stored at −20 C for further analysis. The level of sAA activity was measured through spectrophotometry and salivary protein activity was measured by using the enzyme-linked immunosorbent assay (ELISA) (ZellBio GmbH kit, Veltlinerweg, Germany).

### 2.4. Dietary Sugar Intake and Oral Hygiene Care

A self-administered structured questionnaire was completed by parents/caretakers about the demographic factors and oral hygiene care (frequency of tooth brushing per day), as well as the sweet snacks frequency (>5 times: high risk, 3 to 5 times: moderate risk, <3 times: low risk), stickiness (low: consuming fruit juice and sugar liquids, medium: consuming cake and biscuit, high: Pastel and toffee), and time (between the main meals or along with the main meals) of consuming.

### 2.5. BMI

The participants' barefoot height and weight was measured with a digital scale and a portable stadiometer, respectively. The BMI was calculated through the standard formula: weight (kg) divided by height (m^2^). In order to appropriately compare the subjects of different ages and sexes, the BMI was converted to BMI z-scores and percentile data by using an online calculator (Health Watch Pro software; Version 3.1). Based on the input information of age, sex, weight, height and race, the participants were grouped as follows:

- Underweight: BMI-for-age <5^th^ percentile
- Normal: 5^th^ percentile ≤BMI-for-age <85^th^ percentile
- At risk of being overweight and overweight: 85^th^ percentile ≤BMI-for-age <95^th^ percentile, BMI-for-age >95^th^ percentile

### 2.6. Statistical Analysis

For the statistical analysis, dental caries was considered as the dependent variable; while, the maternal working status (working or non-working), parental educational level, oral hygiene status (tooth brushing per day), snacking frequency, time and stickiness, BMI, and sAA activity were the independent variables. Quantitative data like sAA activity were expressed as mean and standard deviation. Normal distribution of variables was tested with Kolmogorov–Smirnov test. To analyze the questionnaire responses, descriptive statistics were calculated for all variables, and the independent variables were dichotomized and tested with chi-square test to investigate possible associations between the dependent and independent variables. Finally, a backward stepwise regression was performed to identify the predictors of ECC development. Logistic regression analysis and relative risk were expressed as odds ratios with their respective 95% confidence intervals. The statistical analyses were conducted by using SPSS software (version 16.0, SPSS Inc., IL, Chicago, USA) (*α* = 0.05 in all tests).

## 3. Results

In this study, 38 cases of ECC were compared with 41 caries-free controls.  Table 1 displays the distribution of the participants' general characteristics. Among the demographic characteristics, only the mothers' education level was significantly associated with the occurrence of ECC (*P*=0.03) (Table 1). Besides, ECC was found to be significantly correlated with brushing (*P* ≤ 0.001) and all measured dietary habits (*P* ≤ 0.001) (Table 1). The mean BMI was comparable between the ECC (22.12 ± 4.59) and control group (21.39 ± 9.05) with no statistically significant difference (*P*=0.49) (Table 1).

Meanwhile, the mean sAA activity was significantly higher in the control group (*P* ≤ 0.001), indicating it to be inversely related with caries incidence (Table 1). Moreover, a logistic regression analysis was performed to control the variables and identify predictors of ECC. According to the results, sAA activity was shown to be the predictor of ECC development (*P* ≤ 0.001) (Table 2).

## 4. Discussion

ECC is a multifactorial disease associated with multiple variables [18, 28]. One answer for the question that why some children develop ECC while others do not, could be related to the differences between the quantity or quality of salivary components from those children [31]. Salivary alpha-amylase is one of the most important components of the oral fluid, which exhibits various biological functions, related to its high affinity for binding to oral streptococci and carbohydrate digestion [32].

The present study tried to find if ECC is related with sAA enzyme activity, BMI, nutritional and oral hygiene related variables. The sAA activity was found to be significantly higher in caries-free children, indicating an inverse relationship between the ECC and sAA; which was consistent with Borghi et al. findings [21]. Similarly, Scannapieco et al. [33] proposed that sAA could attach to cariogenic bacteria and facilitate their elimination from the oral cavity, and consequently reduce the incidence of ECC.

However, another study reported the mean amylase activity, total protein concentrations, and total IgM to be similar in caries-free and ECC groups. This could be due to their limited sample size (20 in each group) which precluded any significant difference [31].

In contrast to our results, Sitaru et al. [32] detected that caries-active children had higher levels of salivary enzyme activity compared with caries-free groups, and pronounced sAA as a predictive biomarker in preventive dentistry. Their study was conducted on age group of 10 to 14 years old with different criteria for determining caries in comparison with our study which was related to ECC.

Generally, diversity of the findings of the clinical studies in relation to salivary constituents are usually common due to the difficulties in standardizing sampling methods and laboratorial tests [31].

Controversies also exist about the concentration of this protein in saliva. Compared with caries-free controls, higher concentrations of sAA were detected not only in children with ECC [19, 26, 34], but also in caries-susceptible young adults, particularly overweight adolescent girls [10, 27, 35]. Seemingly, excessive amounts of sAA contribute to hydrolysis of starch and acid release by cariogenic bacteria, and thereby raise the risks of dental caries. In contrast, Mojarad et al. [28] concluded that ECC might also be developed in case of decreased sAA concentration. Such a controversy can be justified by the multicomponent nature of human saliva (water, several electrolytes, mucus, glycoproteins, enzymes, and antibacterial compounds), which incorporates a confounding effect that does not allow assessing the effect of a single component in such a media [28].

The function and activity of a specific component in saliva is not necessarily related to its concentration in this media. Furthermore, the concentrations of the salivary constituents are regulated by salivary flow, which is hard to be measured in young children [31]. Thus, based on these controversial results, it would be relevant to investigate how activity of sAA behaves in saliva of children with ECC and if sAA can be considered as a predictor for ECC in preschoolers.

Carbohydrates and sugar are extensively proven as the chief dietary elements that account for the incidence of dental caries [36–39]. Similarly, the current findings confirmed that dental caries are significantly associated with brushing and all dietary habits, particularly the amount and frequency of sugar consumption. The frequency of sugar intake (restricted to main meals or between meals) is reported to play an important role in both dental caries and childhood malnutrition [8]. Moreover, improper feeding practices, lack of parental education, and poor oral hygiene are known to raise the risk of ECC. Oral health is imperative for children to maintain the oral functions such as eating and speech, as well as developing a positive self-image [3].

Pediatric growth disorders have always been a multidisciplinary clinical concern for the specialists, dentists included. Recently, more investigations have been focused on the metabolic effects of obesity on oral health like higher risks of caries and periodontal diseases [12]. Although the relationship between ECC and BMI has been formerly evaluated, the mean BMI, BMI percentiles, or mean weight have been assessed in populations of different ages and mixed sexes [9]. However, in the present study, adjusting the z-scores for both age and sex by Health Watch Pro software yielded more logical report of means.

In line with some previous studies, the current findings revealed the dental caries to be more prevalent among underweight children; however, this was not statistically significant. Kumar et al. [11] noted that the socioeconomic level affected the association between BMI and dental caries. Accordingly, overweight children of high socioeconomic families had less dental caries than the normal-weight children.

Conflicting results have been obtained regarding the relationship between dental caries and BMI in children [7]. While some studies rejected any association between dental caries and obesity [7, 38, 40, 41], a systematic review provided evidence that dental caries is relatable to both low and high BMI [42]. Unlike the present study, some studies noted the dental caries to be more frequent in obese children (BMI >30) than those with normal body weight (BMI <25) [12, 17, 43, 44]. Pannunzio et al. [45] attributed the higher prevalence of caries in obese children to the decreased activity of salivary peroxidase enzyme, which accounts for the antibacterial and antioxidant features of saliva.

It is recommended to emphasize the importance of oral hygiene provide nutritional counseling and take appropriate preventive measures for the children with nutritional imbalance leading to abnormal BMI. Both malnutrition and dental caries can have lifelong negative repercussions for children. An interdisciplinary approach between the pedodontists and primary health care providers or pediatricians can offer a good opportunity to prevent chronic oral diseases and treat these childhood diseases [46].

Due to the difficulties related to the Covid-19 pandemic, it was not feasible to match predisposing factors at case selection stage. However, the groups were almost similar regarding the general characteristics such as sex, age, and parents' education level. Moreover, the situation limited measuring sAA activity at follow-ups to determine how this enzyme changes according to the treatment of caries or caries progression as the child gets older. Therefore, more clinical studies with larger sample sizes, considering matching of the possible confounding variables and evaluating more biochemical parameters are recommended to assess the correlation between sAA and ECC more precisely and predict the potential factors that affect the initiation and development of dental caries in children.

## 5. Conclusion

With respect to the present findings, it can be concluded that children with inherently lower levels of salivary alpha-amylase activity are more susceptible to dental caries. Additionally, the development of dental caries has a strong inverse relationship with both the amount and frequency of sugar consumption. However, no correlation was detected between dental caries and BMI in the present study.

## Figures and Tables

**Table 1:** Distribution of general characteristics of the case and control groups.

Variable				Groups			
				ECC (*n* = 38)	Control (*n* = 41)	*P* value	Odds ratio (95% confidence interval)
Sex	Male (ref)			16 (50%)	16 (50%)	0.451^a^	1.14 (0.46–2.79)
	Female			22 (46.8%)	25 (53.2%)		

Age (mean ± SD [months])				56.5 ± 10.3	53.1 ± 11.9	0.186^b^	—

BMI	Underweight (<5^th^ percentile)			6 (40%)	9 (60%)	0.199	0.44 (0.11–1.74)
	Normal (5^th^<85^th^ percentile)(ref)			12 (60%)	8 (40%)		—
	At risk of overweight (85^th^ to <95^th^ percentile), overweight (≥ 95^th^ percentile)			19 (48.7%)	20 (51.3%)		0.63 (0.21–1.89)

Maternal working status	Working			21 (51.2%)	20 (44.8%)	0.363	1.3 (0.54–3.14)
	Non-working (ref)			17 (44.7%)	21 (55.3%)		

Education level	Paternal		Academic	18 (40.9%)	26 (59.1%)	0.113	0.52 (0.21–1.28)
			≤High school	20 (59.1%)	15 (42.9%)		
	Maternal		Academic	15 (36.6%)	26 (63.4%)	0.028	0.38 (0.15–0.93)
			≤High school	23 (60.5%)	15 (39.5%)		

Salivary *α*-amylase activity (U/L)				222.8 ± 65.7	363.6 ± 69.6	≤0.001	—

Oral hygiene (brushing per day)			Once or less (ref)	32 (68.1%)	15 (31.9%)	≤0.001	0.11 (0.04–0.32)
			Twice	6 (18.8%)	26 (81.3%)		

Snacking	Time	With main meals (scheduled) (ref)		4 (13.8%)	25 (86.2%)	≤0.001	13.28 (3.96–44.59)
		Between main meals (unscheduled)		34 (68%)	16 (32%)		
	Stickiness	Fruit juice and sugar liquids (ref)		1 (4.8%)	20 (95.2%)	≤0.001	—
		Cake and biscuit		17 (54.8%)	14 (45.2%)		24.29 (2.89–204.22)
		Pastel and toffee		20 (74.1%)	7 (25.9%)		57.14 (6.43–508.06)
	Frequency (per day)	<3 times		5 (13.2%)	33 (86.8%)	≤0.001	—
		3–5 times		20 (74.1%)	7 (25.9%)		18.86 (5.27–67.48)
		>5 times		13 (92.9%)	1 (7.1%)		85.8 (9.13–806.68)

a: chi-square test. b: Mann–Whitney test. ref: reference category.

**Table 2:** 8^th^ step of stepwise logistic regression analysis identifying predictors of ECC.

Predictor variables	Control (reference category) vs. ECC	
	Odds ratio (95% confidence interval)	*P* value
*α*-amylase	0.9 (0.95–0.98)	≤0.0001

## Data Availability

The data used to support the findings of this study are available from the corresponding author upon request.
